# Inclusive leadership and employee job performance: mediation of leader-member exchange and organizational identification

**DOI:** 10.3389/fpsyg.2025.1615144

**Published:** 2025-08-08

**Authors:** Shengnan Wang, Kai Feng, Hongyan Wang, Yongxin Li

**Affiliations:** ^1^Psychological Health Education Centre, Henan University of Animal Husbandry and Economy, Zhengzhou, China; ^2^Institute of Psychology and Behaviour, Henan University, Kaifeng, China

**Keywords:** inclusive leadership, job performance, leader-member exchange, organizational identification, longitudinal design

## Abstract

**Background:**

Previous studies indicated that inclusive leadership is related to a series of positive working outcomes, but the internal acting mechanism remains unclear. This study used a three-month longitudinal design to investigate the acting mechanism of inclusive leadership on individual job performance by introducing leader-member exchange and organizational identification as mediators.

**Methods:**

The data were collected through self-reported questionnaires and a leader-rated survey of 206 employees of a car battery manufacturer in central China.

**Results:**

The results revealed that inclusive leadership is positively associated with job performance, leader-member exchange, and organizational identification. A hierarchical regression and bootstrap analysis indicated that organizational identification and leader-member exchange significantly mediated the relationship between inclusive leadership and job performance, and the structural equation model supported the results.

**Conclusions:**

The findings provide significant lessons for how organizations can gain a competitive advantage in terms of employee performance by fostering inclusive leadership, leader-member exchange, and organizational identification.

## 1 Introduction

The concept of inclusive leadership emerged along with the integration of the world economy and diversification of labor. This leadership style has been regarded as a set of behaviors by which leaders express openness, accessibility, and availability (Carmeli et al., 2010; Hirak et al., 2012; Mitchell et al., 2015) as well as respond to followers' needs for belongingness and uniqueness (Barak, 1999; Shore et al., 2011) during their interactions with employees, so as to enhance the followers' sense of responsibility to the organization and to make them feel esteemed and valued within the work group. Over the last decade, scholars have made good progress in discovering the fundamental ideas of inclusive leadership. Previous studies illustrated that inclusive leadership is not only bound to a series of positive outcomes in the workplace, such as job performance (Mitchell et al., 2015), employee work engagement (Choi et al., 2015), job satisfaction (Brimhall et al., 2014), employee wellbeing (Choi et al., 2017), and organizational justice (Sheehan and Anderson, 2015), but also facilitates positive work-related behaviors such as innovation by employees (Javed et al., 2018a,b, 2017). However, there are still gaps in our understanding of the mediating mechanism between inclusive leadership and its outcomes, which requires further empirical research.

Since the concept of inclusive leadership was introduced in China, it has received plenty of interest from numerous researchers (Gao, 2010; Ma et al., 2015; Tang and Zhang, 2015). Initially, research has mostly remained at the level of theoretical discussions (Ma et al., 2015) and reviews (Tang and Zhang, 2015), while recently there has increased number of empirical studies (Ma et al., 2019; Tang et al., 2018; Zhong et al., 2019). Since the concept of inclusive leadership was introduced in China, it has received plenty of interest from numerous researchers (Gao, 2010; Ma et al., 2015; Tang and Zhang, 2015). Initially, research has mostly remained at the level of theoretical discussions (Ma et al., 2015) and reviews (Tang and Zhang, 2015), while recently there has increased number of empirical studies (Ma et al., 2019; Tang et al., 2018; Zhong et al., 2019). Recent years have witnessed a surge in empirical investigations across various Chinese organizational contexts, including healthcare (Zhang et al., 2022; Ahmed et al., 2021), education (Li and Zhou, 2023; Liu and Wu, 2023), and hospitality sectors (Lv et al., 2022; Shen et al., 2025), demonstrating the growing practical importance of inclusive leadership in Chinese organizations. However, since inclusive leadership is highly consistent with Chinese traditional culture and the inclusive perspective of development (Tang and Zhang, 2015), and Chinese organizations are increasingly involved in globalization, leaders are challenged by an increasingly diversified workforce. Therefore, more empirical research on inclusive leadership and its acting mechanism on outcomes is needed to improve leaders' efficiency and effectiveness as well as organizational competitiveness.

This study is theoretically grounded in Conservation of Resources (COR) Theory, which posits that individuals are motivated to acquire, protect, and maintain resources (Hobfoll and Shirom, 2001). When resources are lost or threatened, individuals experience stress and take action to restore resource balance. As a relational leadership style, inclusive leadership serves as a resource provider, helping employees accumulate psychological resources through social support, emotional connection, and development opportunities. COR theory explains why inclusive leadership operates through specific mediating mechanisms. High-quality Leader-Member Exchange (LMX) relationships represent increased leader support and resource allocation to employees, while organizational identification reflects employees' emotional belonging and psychological attachment to the organization. Both mechanisms strengthen employees' resource reserves, which COR theory suggests will be translated into improved work performance over time. Furthermore, COR theory emphasizes that resource accumulation is a dynamic process requiring time to develop, which supports our longitudinal research design spanning 3 months.

High-quality Leader-Member Exchange (LMX) relationships signify greater leader support and resource allocation to employees, while organizational identification (OI) reflects employee emotional belonging to the organization. Both mechanisms strengthen employee resource reserves, which are then translated into work performance (Katsaros, 2024). Furthermore, COR theory emphasizes the dynamic nature of resource accumulation, asserting that time is required for resources to develop (Halbesleben et al., 2014). The psychological impact of inclusive leadership on employees takes time to manifest as behavioral outcomes. For instance, initiatives implemented at Time 1 (e.g., trust-building and tolerance for mistakes) may enhance employee trust through LMX and strengthen OI at Time 2, ultimately driving performance improvements at Time 3.

This study integrates Conservation of Resources (COR) Theory with Social Exchange Theory to provide a comprehensive theoretical foundation for understanding inclusive leadership mechanisms. COR Theory (Hobfoll, 1989) explains the resource-building process through which inclusive leadership operates, while Social Exchange Theory (Blau, 1964) illuminates the relational mechanisms that translate leadership behaviors into employee outcomes. According to COR Theory, individuals strive to obtain, retain, and protect resources, and the accumulation of resources leads to positive outcomes. Inclusive leadership behaviors—openness, accessibility, and availability—function as resource investments that provide employees with psychological resources including social support, recognition, and development opportunities. These resources accumulate over time and are subsequently invested in improved job performance. Social Exchange Theory complements this resource perspective by explaining how the quality of leader-member relationships influences the resource exchange process. When leaders demonstrate inclusive behaviors, they signal their investment in the relationship, prompting employees to reciprocate through enhanced effort and commitment. This theoretical integration explains why inclusive leadership operates through both relationship quality (LMX) and organizational attachment (organizational identification) pathways. The temporal aspect of resource accumulation emphasized in COR Theory supports our longitudinal design, as the theory suggests that resource gains require time to translate into behavioral outcomes. Furthermore, these theoretical mechanisms are particularly relevant in Chinese cultural contexts, where relationship-based interactions and collective identity formation are fundamental to workplace dynamics.

**Figure 1:**
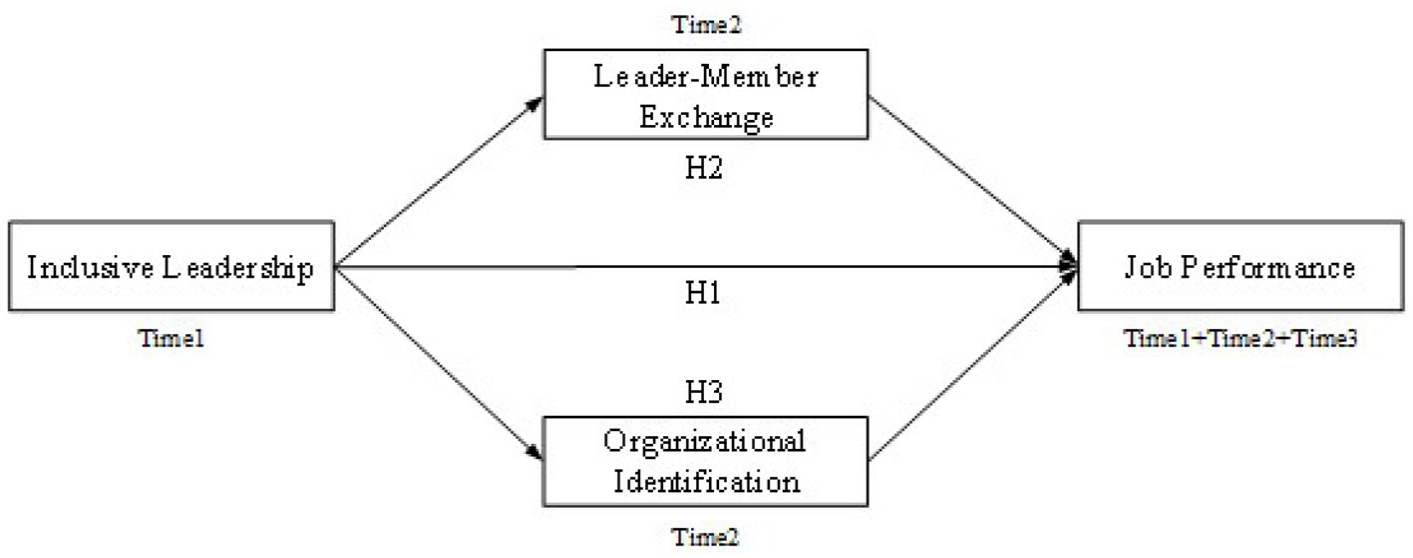
The hypothesized model.

Therefore, this study focused on LMX and OI as possible mediators. It explored whether and what kind of relationship exists between inclusive leadership and job performance, using a longitudinal study of 206 employees of a battery manufacturer over 3 months. The conceptual model is illustrated in Figure 1.

## 2 Theory and hypotheses

### 2.1 Inclusive leadership and employee job performance

Inclusive leadership has been regarded as a mode of relational leadership in which the focus is on leaders listening and paying attention to followers' needs and followers perceiving that leaders are available to them (Hollander, 2012). Inclusive leaders respect employees, treat them fairly, and show tolerance and patience around employee failures. This fosters increasing employee loyalty toward the organization as well as good relationships between employees, which ultimately benefits the organization due to employee improved job performance (Boehm et al., 2014). Empirical studies have confirmed a positive relationship between inclusive leadership with some outcomes. For example, inclusive leadership could positively predict employee engagement and is correlated with employee cognition of psychological safety (Carmeli et al., 2010; Javed et al., 2017). It also has a positive influence on productivity, employee innovative work behavior (Javed et al., 2018a,b), learning from errors (Ye et al., 2018), work engagement (Choi et al., 2015), and retention rate (Ma et al., 2014).

In China, in a study of college teachers, Fang and Jin (2014) found that the leader's inclusion of employees significantly affected their team performance. Cheng (2014) reported a positive correlation between inclusive leadership and psychological ownership and team cohesiveness, as well as negative correlations with turnover intention, counterproductive behavior, and cynicism. Ma et al. (2019) conducted an empirical study on medical teams and confirmed the positive impact of inclusive leadership on team performance. However, most previous studies are based on self-reported measurements of performance variables (Fang and Jin, 2014; Ma et al., 2019). In this study, other-rated data on job performance were collected to further investigate inclusive leadership and generate a comprehensive understanding of the concept. We hypothesize the following:

**Hypothesis 1**. Inclusive leadership is positively related to job performance.

### 2.2 Mediating effects of leader-member exchange

The LMX theory developed by Graen and Uhl-Bien (1995) explains how leaders treat subordinates distinctively. It posits that the degree of affinity between leaders and their subordinates is an important variable affecting performance. According to Liden and Maslyn (1998), LMX consists of four conditions: loyalty, affect, contribution, and specialized admiration. This characterizes high-quality and low-quality “leader-member” relationships in terms of social support, communication, individual contribution, and materials constraints. In a high-quality leader-member relationship, employees experience a high level of support and trust from their immediate leader and consequently enjoy a familiar relationship with the leader (Wayne et al., 1997).

Inclusive leadership is a type of relational leadership (Hollander, 2012), that inevitably involves the leader-member relationship. This relational aspect is particularly significant in Chinese organizational contexts, where guanxi (relationship networks) and high-quality interpersonal connections are culturally valued. Recent studies have consistently demonstrated the critical role of leader-member relationships in Chinese organizations across various sectors (Xie et al., 2023; Wang et al., 2024; Cheng et al., 2024). The inclusiveness of leaders is always reflected in their interaction with subordinates. Javed et al. (2018a) argued that inclusive leadership facilitates a relationship of social interaction between leaders and followers in which both parties trust and support each other as a team and both enjoy a high-quality relationship of open communication, confidence, consideration, maturity, and effective interaction (Maslyn and Uhl-Bien, 2001; Sullivan et al., 2003). This is based on the social exchange theory that individuals interact and maintain relationships with others in the expectation of outcomes and returns (Blau, 1968; Homans, 1958). As a high degree of inclusive leadership may give employees a good perception of LMX (Hollander, 2012), when LMX is high, the level of social exchange will be high, which will subsequently enhance the trust and esteem between leaders and subordinates; this, in turn, may accelerate effective interactions within the group and motivate the members to accomplish additional achievements. On the contrary, when LMX is low, the leader can only expect normal job performance from subordinates by exercising their official authority and distributing standard benefits. Studies have shown that high-quality LMX could enhance employee sense of responsibility (Langfred and Moye, 2004) and thus improve their job performance (Kalbers and Cenker, 2008). Early studies verified that LMX was significantly correlated with superiors' evaluation of subordinates' performance (Gerstner and Day, 1997), as well as the job performance of group members. Employee performance in cases of high-quality LMX was 20% better than in low-quality LMX cases (Mayfield and Mayfield, 1998).

As inclusive leadership focuses on the relationship between leaders and members, while LMX can effectively predict individual job performance, LMX may play a mediating role between inclusive leadership and employee job outcomes. Javed et al. (2018b), through a study of small Pakistani textile companies, showed that inclusive leadership promoted innovative work behaviors via the mediation of LMX. Recent Chinese research has provided substantial evidence for LMX mediation mechanisms. Xie et al. (2023) found that leader-member exchange played a crucial role in mitigating work-related stress among Chinese nurses, demonstrating the importance of high-quality leader-member relationships in Chinese healthcare contexts. Similarly, Cheng et al. (2024) confirmed that leader-member exchange was significantly associated with nurses' innovative behaviors in Chinese hospitals, indicating that LMX quality is fundamental to positive outcomes in Chinese organizational settings. Furthermore, Wang et al. (2024) demonstrated that leader-member exchange moderated the relationship between leadership and psychological safety in Chinese contexts, while Arshad et al. (2024) found that LMX effectiveness was particularly pronounced in Chinese organizational environments compared to other cultural contexts. These recent findings support the critical role of LMX in Chinese organizations and justify our focus on this mediating mechanism. Hence, combining with Hypothesis 1, we hypothesize the following:

**Hypothesis 2**. LMX mediates the relationship between inclusive leadership and job performance.

### 2.3 Mediating effects of organizational identification

The concept of OI originated from social identity theory (Tajfel, 1972), which refers to the consistency of members' behaviors and concept with the organization they belong to (Mael and Ashforth, 1992). The members agree that they have rational contracts with and responsibilities to the organization as well as an irrational sense of belongingness and dependence. The behavioral results of their dedication to organizational activities are dominant on this psychological basis (Jian et al., 2017). These characteristics of the concept could be used as a mediator between organizational goals and organizational activities; that is, OI has an impact on organizational operation activities and thus might advance the achievement of organizational goals.

In order to improve employee perception of OI in organizational practices, scholars have made good progress in the study of the relationships between OI and its antecedents. The empirical evidence demonstrates that a series of organizational variables are associated with employee perception of OI, such as organizational culture, organizational competition, organizational support, organizational justice, and organizational communication (Li, 2011, p. 24–28). As leaders usually occupy vital positions in organizations, and to a large extent determine the organizational activities and development (Kim and Yukl, 1998), being the substitutes for and representatives of organizations, the individual's perception of the organization is largely influenced by interaction with the leader. Studies have shown that a series of different leadership styles, such as transformational leadership and transactional leadership (Epitropaki and Martin, 2004; Wang et al., 2013), spiritual leadership and authentic leadership (Wang et al., 2013), and paternalistic leadership (Wu et al., 2018) have predictive effects on individual OI.

Differing from traditional leadership theories, inclusive leadership places the emphasis on employee perception of whether they have been accepted and respected by their colleagues through the experience of whether their psychological demands have been satisfied (Shore et al., 2011). This emphasis on acceptance and belonging is particularly resonant in Chinese collectivistic culture, where group harmony and organizational identification are highly valued (Liu et al., 2021; Lv et al., 2022). This would subsequently lead to employee acceptance of, recognition of, and identification with the organization (Randel et al., 2018). Despite this, as inclusive leadership and OI share the same theoretical origin—the social identity theory—inclusive leaders may promote employee OI by influencing their acceptance of organizational goals and constructing a favorable working environment. By nature, inclusive leaders are accessible and listen to their followers, which may expedite employee sharing of the goals and values of the organization. Tang et al. (2018) proved that inclusive leadership could reinforce employee wellbeing via the mediating role of employee goal acceptance. Besides, the inclusive leaders' availability and openness help establish an effective communication atmosphere and cooperative work climate, which has been reported to positively predict employee OI (Mael and Ashforth, 1992).

Considering the conception of inclusive leadership, it could be expected to facilitate an improvement in individual OI. Previous studies have provided huge amounts of evidence for OI having a positive relationship with a series of attitudinal and behavioral outcome variables, such as intention to resign, affective commitment, work involvement, and organizational citizenship behavior (see Li, 2011, p. 33–40). Thus, combining with Hypothesis 1, we hypothesize the following:

**Hypothesis 3**. OI mediates the relationship between inclusive leadership and job performance.

## 3 Materials and methods

### 3.1 Samples and procedures

Participants were chosen from a car battery manufacturer in Henan Province, China through convenience sampling. Before administering the survey, we explained the research purpose and the confidentiality principle to all the participants and guaranteed them the right to withdraw from the study at any time. The surveys were conducted in each department with consent from all the participants. We distributed questionnaires to 206 employees and received 176 sets of matched valid responses. Of the participants, 116 (65.9%) were male while 60 (34.1%) were female. In terms of age, 33 participants (18.75%) were 25 years old or younger, 124 (70.45%) were aged 26–35 years, and 19 (10.8%) were 36 years old or older. Eighty-two participants (46.6%) were married and 94 (53.4%) were not. As for educational level, 58 (33%) participants had college degrees or lower, while 118 (67%) participants had a bachelor's degrees or higher. Regarding tenure, 132 (75%) participants had worked there for 5 years or less, and 44 (25%) participants had worked for 6 years or more.

Data were collected via face-to-face questionnaire surveys at three time points over the 3 months. We provided an identification number to each participant and asked each of them to indicate his or her own number on the questionnaires for later matching. At Time 1, employees were asked to complete the demographic information forms and to rate their perception of their direct leaders' inclusive leadership. A month later, at Time 2, we started the second survey to measure the LMX and OI of the employees. Lastly, the performance scores of the employees, as assigned by their direct leaders, in the 3 months of Time 1, Time 2, and Time 3 were calculated. In the first two surveys, 412 questionnaires were issued and 385 were received, with a response rate of 93.4%. To match the job performance data that were collected at the three stages with the participant's identification number, some invalid questionnaires and unmatched answers were removed. The remaining 352 valid responses were finally sorted into 176 sets of questionnaires, with a response rate of 85.4%.

### 3.2 Measures

#### 3.2.1 Inclusive leadership

The employee perception of inclusive leadership was measured by Carmeli et al.'s (2010) inclusive leadership scale. The scale consists of three dimensions with nine items: openness (three items, e.g., “The manager is open to hearing new ideas”), availability (four items, e.g., “The manager is available for consultation on problems”), and accessibility (two items, e.g., “The manager is accessible for discussing emerging problems”). All the items were scored on a 5-point Likert scale (1 = not at all to 5 = to a large extent) and the summation range was 9–45. Higher scores indicated that subordinates perceived more inclusiveness from their leaders. The Cronbach's alpha was 0.91.

#### 3.2.2 Leader-member exchange

The leader-member exchange scale compiled by Graen and Uhl-Bien (1995) was used to measure LMX as perceived by the employees. The scale includes seven items, such as “How well does your leader understand your work problems and needs?” and “How well does your leader recognize your potential?” All the items were scored on a 7-point Likert scale under positive direction (ranging from 1 = not at all/none to 7 = very high/fully). Higher scores indicate a higher quality of the LMX relationship. The Cronbach's alpha was 0.91.

#### 3.2.3 Organizational identification

The organizational identification scale developed by Mael and Ashforth (1992) was selected to measure OI. There were six items (e.g., “The organization's successes are my successes”) positively scored on a 5-point Likert scale (1 = strongly disagree; 5 = strongly agree). Higher scores represent a higher level of OI. The Cronbach's alpha was 0.90.

#### 3.2.4 Employee job performance

To evaluate the employee job performance, we adopted the self-designed performance evaluation method of the enterprise. The enterprise has a mature and advanced management system that enables it to improve constantly to make changes to meet market requirements along with its own characteristics. The job performance evaluation form used in this study is the enterprise's current version, which has been revised several times and used for years with good results. The assessment form is marked using a centesimal system and is scored directly by the employee superiors. It has four parts—assessment of employee work attitude (20 points), collaborative ability (25 points), work execution ability (50 points), and self-improvement ability (5 points)—and each part has specific subdivisions. The final total score of each employee is converted into a performance score and then again to determine the performance coefficient. The results of the department's performance coefficient are finalized through a review by the salary performance administrators and the approval of the general manager of the integrated management department. This study used the original performance score as the basis for the evaluation of an employee's job performance. The mean of the three job performance scores over the 3 months was calculated as the indicator of the outcome variables in the present study.

### 3.3 Data analysis

This study employed SPSS 25.0 and AMOS 23.0 analysis software as well as deviation-correction bootstrap technology for the data analyses, which was done in four steps. First, the descriptive statistics was conducted and the Pearson correlation analysis applied to test the correlation between the variables. Second, an SPSS hierarchical regression analysis was conducted to investigate the mediating effects of LMX and OI on the relationship between inclusive leadership and employee job performance. Third, the deviation-correction bootstrap method was adopted to further examine the mediating role of LMX and OI. Finally, AMOS 23.0 was used to verify the hypothesis model with an unbiased estimation method.

## 4 Results

### 4.1 Descriptions and correlations

Table 1 presents the demographic characteristics of the study participants. The sample consisted of 176 employees from a car battery manufacturer, with males comprising the majority (65.9%). Most participants were in the 26–35 age range (70.4%), slightly more were unmarried (53.4%), and the majority held bachelor's degrees or higher (67.0%). In terms of organizational tenure, three-quarters of participants (75.0%) had worked at the organization for 5 years or less.

**Table 1:** Demographic characteristics of participants (*N* = 176).

**Variable**	**Category**	**Frequency**	**Percentage**
**Gender**			
	Male	116	0.659
	Female	60	0.341
**Age**			
	25 years or younger	33	0.188
	26–35 years	124	0.704
	36 years or older	19	0.108
**Marital status**			
	Married	83	0.472
	Unmarried	88	0.5
	Divorced	5	0.028
**Educational level**			
	College or below	58	0.33
	Bachelor's or above	118	0.67
**Tenure**			
	5 years or less	132	0.75
	6 years or more	44	0.25

Table 2 presents the descriptive statistics and correlations among the main study variables: inclusive leadership, LMX, organizational identification, and job performance. The mean score for inclusive leadership was 3.81 (SD = 0.69), which was notably higher than the theoretical midpoint of 3.0 on the 5-point Likert scale, indicating that employees generally perceived their leaders as inclusive. Similarly, the mean scores for LMX (M = 4.44, SD = 0.92) and organizational identification (M = 3.74, SD = 0.76) were both above their respective theoretical midpoints.

**Table 2:** Correlations among study variables.

**Variables**	**M ± SD**	**1**	**2**	**3**	**4**
1. IL	3.81 ± 0.69	1			
2. OI	3.74 ± 0.76	0.725^**^	1		
3. LMX	4.44 ± 0.92	0.809^**^	0.700^**^	1	
4. JP	78.31 ± 6.75	0.487^**^	0.474^**^	0.550^**^	1

*N* = 176; IL, Inclusive Leadership; LMX, Leader-member Exchange; OI, Organizational Identification; JP, Job Performance. ^*^p < 0.05, ^**^p < 0.01, ^***^p < 0.001.

Correlation analyses revealed significant positive relationships among all main variables. Inclusive leadership demonstrated a strong positive correlation with job performance (*r* = 0.487, *p* < 0.01), providing initial support for Hypothesis 1. LMX showed the strongest correlation with job performance (*r* = 0.550, *p* < 0.01), while organizational identification also exhibited a substantial positive relationship with job performance (*r* = 0.474, *p* < 0.01). These correlation patterns provide preliminary evidence for the proposed mediation relationships.

Regarding demographic differences in inclusive leadership perceptions, ANOVA and *t*-test analyses revealed that educational level was the only demographic variable that significantly influenced perceptions of inclusive leadership (*F* = 4.09, *p* = 0.008). No significant differences were found for gender (*t* = 0.812, *p* = 0.369) or age (*F* = 1.217, *p* = 0.305), suggesting that inclusive leadership perceptions were relatively consistent across these demographic categories. Therefore, Hypothesis 1 was supported by the significant positive correlation between inclusive leadership and job performance.

The measurement model—which allowed every item to load on its respective construct—was compared with two nested models. The results of the confirmatory factor analyses are given in Table 3.

**Table 3:** Measurement model.

**Model**	**χ^2^**	**df**	**RMSEA**	**CFI**	**RMR**
3-factor model (the measure model)	454.576	196	0.087	0.913	0.066
2-factor model (combined with IL and LMX)	551.030	198	0.101	0.881	0.059
1-factor model (all items loading on a single factor)	686.959	199	0.118	0.836	0.064

As shown, the three-factor measurement model (inclusive leadership, organizational identity, and LMX) had a better fit than the other models. Combined with inclusive leadership and LMX, or by setting all item loadings on one factor, the model did not fit well. This indicated that the construct validity was acceptable. Even though inclusive leadership, organizational identity, and LMX were correlated with each other, these variables need to be analyzed separately.

### 4.2 Hierarchical regression analysis

**Figure 2:**
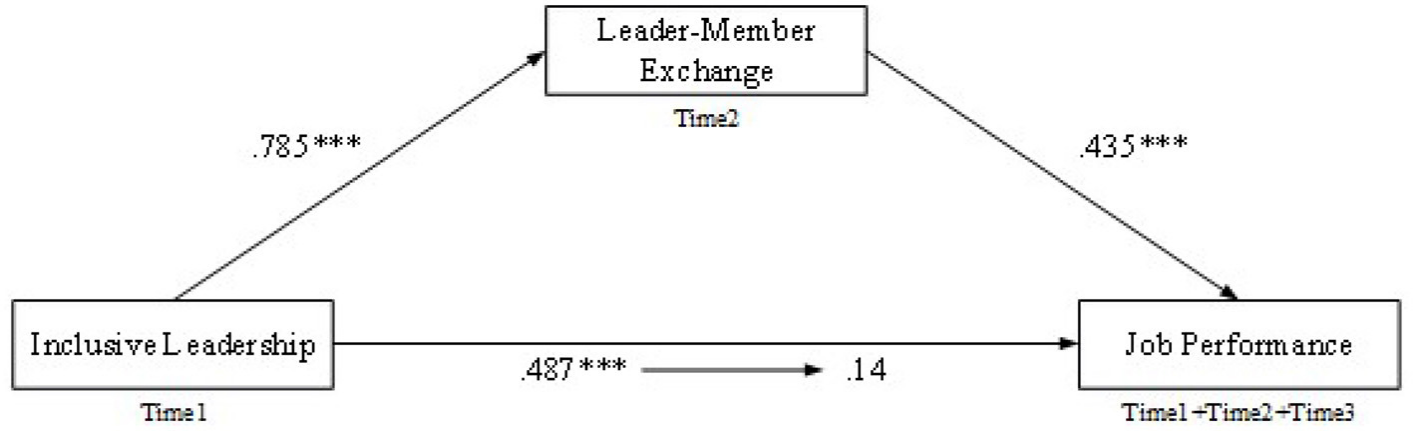
The mediation model of leader-member exchange. ***p < 0.001.

**Figure 3:**
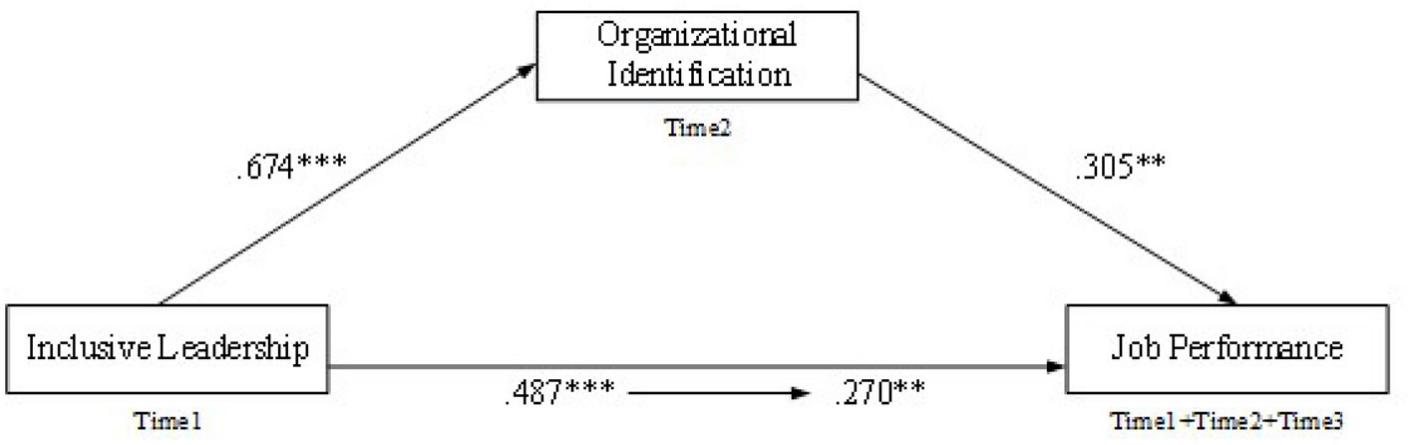
The mediation model of organizational identification. **p < 0.01, ***p < 0.001.

Initially, Table 4 revealed the results of the mediating roles of LMX and OI in the relationship between inclusive leadership and employee performance that were analyzed by applying the SPSS hierarchical regression technique. First, we entered the control variables of gender, age, marital status, educational level, and tenure; second, we input the independent variable of inclusive leadership; third, we entered LMX and OI. As shown in Table 4, the impact of inclusive leadership on job performance (β = 0.49, *p* < 0.001) was significant at the level of 0.01. However, after we entered LMX, it decreased from 0.49 to 0.14 (not significant), which implied there was a complete mediation between inclusive leadership and job performance (for more details see Figure 2). While, after entering OI, the impact decreased to 0.270, but was still significant at a level of 0.01, which illustrated there was a partial mediating effect in the relationship between inclusive leadership and job performance (for more details see Figure 3). Therefore, Hypothesis 2 and Hypothesis 3 were preliminarily confirmed.

**Table 4:** Results of hierarchical regression analysis of study variables.

**Variable**	**Job performance (LMX)**			**Job performance (OI)**		
Gender	0.029	0.048	0.023	0.029	0.048	0.055
Dummy 1	0.118	0.077	0.063	0.118	0.077	0.115
Dummy 2	−0.052	0.036	0.036	−0.052	0.036	−0.003
Marital status	0.193^*^	0.156^*^	0.135	0.193^*^	0.156^*^	0.162^*^
Educational level	−0.125	0.003	0.034	−0.125	0.003	0.022
Tenure	0.054	0.047	0.029	0.054	0.047	0.096
IL		0.487^***^	0.140		0.487^***^	0.270^**^
OI						0.305^**^
LMX			0.435^***^			
*R* ^2^	0.047	0.264	0.327	0.047	0.264	0.304
*F*	1.381	8.614^***^	10.159^***^	1.381	8.614^***^	9.129^***^
Δ*R*^2^	0.047	0.217	0.063^***^	0.047	0.217	0.040
Δ*F*	1.381	49.622^***^	15.699^***^	1.381	49.622^***^	9.648^**^

*N* = 176. Coding for categorical variables: Gender (1 = male, 0 = female); Age Dummy 1 (1 = 25 years or younger, 0 = 26 years or older); Age Dummy 2 (1 = 35 years or younger, 0 = 36 years or older); Marital Status (1 = married, 0 = unmarried); Educational Level (1 = college degree or below, 0 = bachelor's degree or above); Tenure (1 = 5 years or less, 0 = 6 years or more). IL, Inclusive Leadership; LMX, Leader-member Exchange; OI, Organizational Identification. ^*^p < 0.05, ^**^p < 0.01, ^***^p < 0.001.

Then, a bootstrapping analysis with 5,000 iterations was adopted to further investigate the respective roles played by LMX and OI in the relationship between inclusive leadership and employee performance. As expected, the results provided robust evidence to support the mediators (Hypothesis 2 and Hypothesis 3). In detail, the 90% bias-corrected confidence interval for the indirect effect of LMX excluded zero [*CI* = (0.161, 0.597)], which indicated that the mediating effect of LMX was significant, and the mediated effect was 0.379. Likewise, the 90% bias-corrected confidence interval for the indirect effect of OI did not include zero either [*CI* = (0.036, 0.463)], which supported the significant mediati effect of OI, and the mediated effect was 0.250. A further test examined the discrepancies between the two mediators, while the result demonstrated that the differences were not significant (*T* = −0.504, *p* = 0.614).

### 4.3 Structural equation model

**Figure 4:**
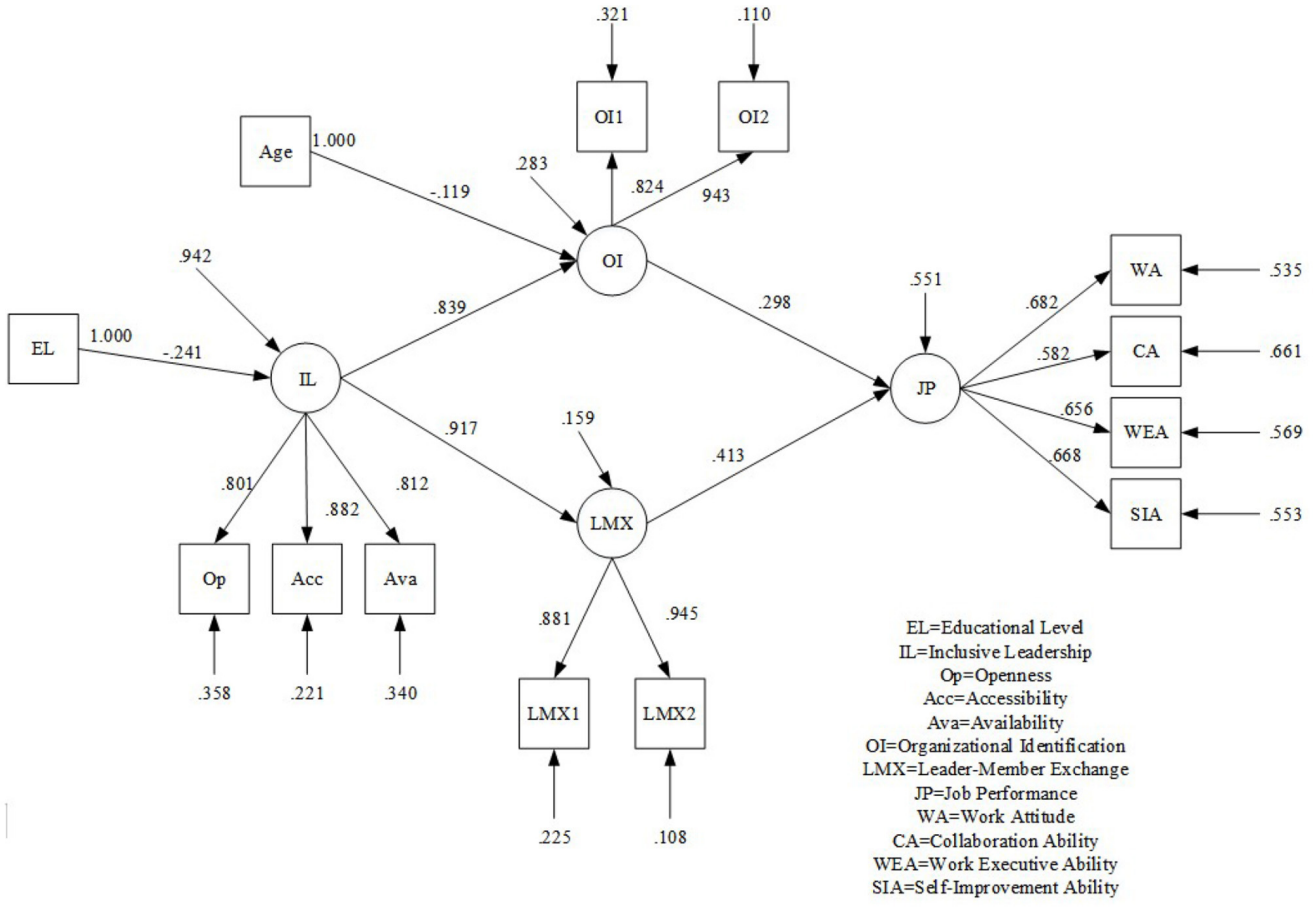
An intermediary model of inclusive leadership influencing employee performance.

The results from the SPSS hierarchical regression analysis demonstrated that LMX and OI both mediated the relationship between inclusive leadership and employee job performance. After that, we used AMOS 23.0 to further test the hypothesized model with an unbiased estimation method. Inclusive leadership took its three dimensions as explicit variables, LMX and OI were cross-packaged according to the load of their items, which were two explicit variables, and its four dimensions as explicit variables verified job performance. The results are shown in Figure 4. Afterwards, when the direct impact of inclusive leadership on job performance was added, the path coefficient was β = −0.023, *p* = 0.949, which was not significant. The fitting indices of the full mediation model and the partial mediation model are shown in Table 5, which proved the full intermediary role of LMX and OI between inclusive leadership and employee job performance. Hence, Hypothesis 2 and Hypothesis 3 were supported.

**Table 5:** Results of structural equation model (*N* = 176).

**Model**	**χ^2^**	**df**	**χ^2^/df**	**RMSEA**	**TLI**	**CFI**	**SRMR**
Full mediation	118.592	61	1.944	0.073	0.942	0.954	0.056
Partial mediation	118.587	60	1.976	0.074	0.940	0.953	0.056

## 5 Discussion

This three-month longitudinal study examined the mechanisms through which inclusive leadership influences job performance in a Chinese manufacturing context. Our findings provide strong empirical support for a dual mediation model in which both leader-member exchange (LMX) and organizational identification (OI) serve as significant mediators. The results demonstrate that inclusive leadership operates through both relational (LMX) and identificational (OI) pathways, consistent with our integrated theoretical framework combining COR Theory and Social Exchange Theory. The LMX pathway reflects the resource exchange process predicted by Social Exchange Theory, while the organizational identification pathway represents the resource accumulation process described by COR Theory.

In this study, we investigated the relationship between inclusive leadership and employee job performance, examining LMX and OI as mediating variables. Our findings contribute to the growing body of recent Chinese research on inclusive leadership (Li and Zhou, 2023; Liu and Wu, 2023; Wu and Li, 2023; Man and Fan, 2025) while extending this research into the manufacturing sector with a longitudinal design. Valid data were collected from 176 employees of a car battery manufacturer in central China via a three-stage longitudinal research design. The study results proved the mediating effects of LMX and OI on the impact of inclusive leadership on employee job performance.

The mediating mechanisms identified in our study—LMX and organizational identification—are particularly meaningful when examined within China's distinctive cultural framework. Chinese culture, characterized by high collectivism, power distance, and relationship orientation (Hofstede, 2001), creates unique conditions that amplify the effectiveness of inclusive leadership through these specific pathways. The dual mediation model revealed in our study reflects fundamental aspects of Chinese workplace dynamics. In Chinese organizations, employee performance is not merely a function of individual capabilities or task requirements, but is deeply embedded in social relationships (guanxi) and collective identity formation. Our findings demonstrate that inclusive leadership operates through these culturally salient mechanisms to enhance performance outcomes.

As shown by the descriptive and correlative analysis, inclusive leadership demonstrates non-significant differences in the selected demographic variables, except for educational background. The results differ from previous studies that claimed that factors such as age, gender, and education level could predict differences in individual inclusive perception (Sessler Bernstein and Bilimoria, 2013; Cho and Mor Barak, 2008; Findler et al., 2007). For example, Mor Barak et al. (1998) found that men and white people had reported perceiving more inclusiveness from their leaders compared to other populations in a study on the electronics industry. After eliminating the group differences between subjects, the inconsistency may be attributed to the cultural differences between the East and the West, as individuals may acquire distinctive understandings of the concept of inclusive leadership in diversified working contexts. Moreover, the government's support and the development of the social economy in China empowered women having equal rights with men to be educated, employed, and promoted and that may enable them to perceive a stronger sense of being held in esteem and valued by the organization they joined. Reports by the US Census Bureau indicated that in China, the labor force participation rate of women was ~70% and ranked first in the world, and 37% of the management in organizations are women, which ranked second in Asia (Hays Asia Salary Guide, 2018). Therefore, it is understandable why there is no gender differences in employee perception of inclusive leadership in this study.

Regarding the inconsistency in age difference, due to the fact that the participants in this study are employees at a young enterprise, and the age range of the majority of middle managers and ordinary employees was from 25 to 35. The age distribution may provide an explanation of the results. However, as this phenomenon widely exists in small and medium enterprises, our results are significant and make both theoretical and practical contributions.

Lastly, employee perception of inclusive leadership is significantly different depending on their level of education, which is in accordance with the reality of the actual management activities of enterprises. Due to a fiercely competitive and turbulent environment, the higher qualified and skilled employees play an increasingly essential role in the enterprise. As their attributes are part of the core competitiveness of an organization, these talents are bound to be valued and used repetitively. Generally, they have better opportunities to become in-group members and to have closer relationships with the leaders. With the enhancement of the relationship, the perceived degree of inclusive leadership will be improved automatically. However, employees with a lower educational background are mostly at the grassroots level of the enterprise, which is why managers pay comparatively little attention to them. Furthermore, their communication with leaders is mostly focused on work tasks, which may result in them perceiving a lower level of inclusiveness from the leaders.

Moreover, the study results enrich the empirical evidence on the predictive impacts of inclusive leadership on employee job performance. This study demonstrates that inclusive leadership can not only predict employee innovation ability (Carmeli et al., 2010), work engagement (Choi et al., 2015), and other specific performance variables, but also have influences on the employee overall job performance in both direct and indirect ways. While job performance is one facet of organizational management outcomes, it reflects the effects of inclusive leadership on organizational performance to some extent. Studies on the measures that leaders could take to encourage employees to voluntarily put in extra work effort is crucial, as employee performance is an indispensable factor that enables organizations to maintain their advantage and competitiveness, cope with emerging challenges, meet market demands, or even cause industrial revolutions (Carmeli et al., 2010).

While our study focuses on manufacturing context, recent research provides evidence for the generalizability of our findings across different industries within China. The LMX and organizational identification pathways identified in our study have been confirmed in various Chinese organizational contexts. For instance, Cheng et al. (2024) found similar LMX mediation effects in Chinese healthcare settings, while Lv et al. (2022) demonstrated organizational identification mediation in Chinese hospitality industry. Furthermore, Zhang et al. (2022) confirmed that inclusive leadership operated through similar psychological mechanisms in Chinese healthcare organizations, and Liu et al. (2023) found comparable mediation patterns among Chinese university teachers. These convergent findings across healthcare, hospitality, and education sectors suggest that our dual mediation model captures fundamental psychological processes that operate consistently across different Chinese organizational contexts. The consistency of findings across industries supports the theoretical validity of our COR-based model while acknowledging that the strength of relationships may vary depending on industry-specific characteristics such as work autonomy, supervision intensity, and team interdependence.

Meanwhile, compared with other leadership theories, inclusive leadership prioritizes openness, accessibility, availability, and responding to followers' psychological needs such as belongingness and uniqueness (Shore et al., 2011; Randel et al., 2018) as the key factors of the theory, which enhances the connotations of how to be an excellent leader. Inclusive leaders prefer to be open-minded to listen to their followers, be approachable to communicate, and accept their followers' ups and downs in work and encourage them to bounce back. This improves their followers' feeling of psychological safety (Carmeli et al., 2010) and trust (Javed et al., 2018b), and consequently makes the leader-member relationship grow closer and establishes a favorable climate in the workplace.

Moreover, the leader's inclusiveness would empower employees to perceive a comparatively high degree of work autonomy and motivate them to reach their full potential. From the perspective of fairness, inclusive leaders tend to hold employees with disparate backgrounds in equal esteem and treat them equally and equitably, providing them with fair opportunities to be promoted, particularly among diversified labor (Ryan, 2007). It is acknowledged that a happy workforce produces more, so companies will eventually earn a good reputation and increase their profits by implementing inclusive leadership.

Besides that, our study contributes to enrich the leadership literature by constructing a mediation model in which introducing LMX and OI as mediators provides an unambiguous understanding on the role of a specific form of relational leadership, i.e., inclusive leadership. We attempted to build on previous research on the role of leadership in fostering employee productivity. In the proposed model, LMX refers to the affective bond of the followers with the leader (Graen and Uhl-Bien, 1995), while OI refers to the degree of employee recognition of organization and leadership at the psychological level (Mael and Ashforth, 1992). Specifically, inclusive leaders respect employees, attach importance to their needs, and recognize their contributions. In this way, they gain their support and respect. During the interaction process, the formation of effective communication and a cooperative atmosphere within the organization is gradually developed, employee sense of belongingness is enhanced, and they regard themselves as the insiders of the work group or the organizations they belong to.

In terms of achieving organizational goals, usually employees often refrain from offering advice due to their concern about the risks associated with voicing concerns that could lead to some uncertain personal consequences, which outweigh the potential benefits to the organization (Milliken et al., 2003). Inclusive leaders empower employees with higher a degree of psychological safety and show a willingness to listen to their views and innovative ideas (Javed et al., 2018a). This is conducive to employees speaking up and advancing their acceptance and recognition of the goals of the organization (Detert and Burris, 2007; Detert and Treviño, 2010). Regarding the process of goal setting, employees with high LMX and OI are likely to regard organizational goals as their personal goals, and therefore are likely to be diligent and enthusiastic to contribute their own efforts to organizational activities. Such a high degree of LMX and OI can not only stimulate employee intrinsic work motivation and drive them to work hard for organizational goals, but also facilitate employee engagement and high-quality self-management, and thus induce a substantial improvement in their job performance (Choi et al., 2015).

The parallel mediation of both LMX and organizational identification reveals a culturally grounded dual-pathway mechanism that is particularly relevant in Chinese organizational contexts. These two mediators operate synergistically, reflecting the complementary cultural dimensions of relationship orientation (guanxi) and collective identity formation that are fundamental to Chinese workplace dynamics.

The simultaneous operation of both mediators suggests that inclusive leadership effectiveness in China requires attention to both interpersonal relationship quality and collective identity formation. This dual requirement reflects the complex nature of Chinese organizational culture, where individual-leader relationships must coexist harmoniously with collective organizational commitment. The cultural concept of “harmony in diversity” (he er bu tong) provides theoretical grounding for understanding why both mediators are necessary in Chinese contexts.

## 6 Implications for practice

Through empirical research on inclusive leadership, this study demonstrates the significance of inclusive leadership for organizations for reaping both tangible and intangible benefits. These findings are particularly relevant given recent developments in Chinese organizational management, where inclusive leadership has been shown to address contemporary challenges such as employee wellbeing during crises (Ahmed et al., 2021), innovation promotion in knowledge-intensive sectors (Zhang et al., 2022), and voice behavior enhancement in educational institutions (Liu and Wu, 2023). First, for organizations, it is advisable to evaluate the inclusiveness of potential leader candidates, particularly in knowledge-intensive organizations. However, to some extent, inclusive leadership is supposed to be a set of organizational behaviors, and it can cultivated and developed with targeted training. Thus, it is strongly recommended that organizations offer training programs for their employees. This would not only benefit organizations in terms of fostering excellent potential leadership talent, but also help the followers to recognize inclusiveness from their leaders.

For leaders, in terms of how to be inclusive, the initial requirements are showing respect for and trust in employees (Ryan, 2006). In practice, it is recommended that leaders show esteem for and recognize employee contributions and avoid taking their diligence and efforts at work for granted. Second, delivering productive feedback on employee performance would be beneficial for them to improve their job-related abilities. Third, being a patient listener would facilitate employee willingness to speak up and enable the leader to receive more innovative ideas.

As the results demonstrate that inclusive leadership improves employee job performance through the mediating action of LMX and OI, leaders are encouraged to increase emotional interactions with employees. For example, to bridge the affective distance between each other, the leader provides effective support when the followers are suffering from frustration at work. In addition, leaders should provide their employees with a supportive, open, and inclusive working climate to reinforce their psychological safety and motivate them to use their individual talents to reach their full potential. If the external environment is appropriate and comfortable, employees could save more energy by focusing on their job and subsequently produce more, rather than having to adjust to adapt to adverse situations.

Our findings demonstrate that inclusive leadership represents a culturally adaptive leadership approach that effectively bridges traditional Chinese values with contemporary organizational needs. The effectiveness of the LMX and organizational identification pathways reflects inclusive leadership's ability to honor traditional Chinese cultural values while addressing modern workplace challenges.

The cultural relevance of our dual mediation model is further supported by the changing nature of Chinese organizations. As Chinese companies become more diverse in terms of generation, education, and geographic background, inclusive leadership provides a culturally sensitive approach to managing this transition by honoring traditional relationship values while fostering inclusive organizational cultures.

## 7 Limitations and future research directions

Based on empirical research, this study used a variety of statistical methods to explore the relationship between inclusive leadership and employee job performance and its acting mechanism. However, this study still has two limitations.

The first limitation is with regards to the sample. This study adopted the principle of convenience sampling, and the respondents are employees from one private enterprise. Even though the distribution of the demographic variables of the sample is relevant to the industry, it may not able to reflect the society as a whole. For example, this study was carried out in a battery manufacturing enterprise, and therefore, male participants accounted for a large proportion of the sample. While our single-organization design limits generalizability, recent cross-industry research in China provides confidence in the broader applicability of our findings. Studies in healthcare (Cheng et al., 2024; Zhang et al., 2022), hospitality (Lv et al., 2022; Siyal et al., 2023), education (Li and Zhou, 2023; Liu et al., 2023), and other service sectors have identified similar mediation mechanisms, suggesting that our theoretical model captures universal psychological processes within Chinese cultural contexts. However, industry-specific factors may influence the relative strength of the mediation pathways. Manufacturing environments with close supervision and team-based work structures may amplify LMX effects, while knowledge-intensive industries might show stronger organizational identification effects due to higher employee autonomy and professional identity considerations. Future research should examine these potential industry moderators systematically. Compared with other occupations, due to a positive correlation between inclusive leadership and educational level, knowledge workers tend to perceive more inclusiveness from their leader. In future, researchers could apply various methods to expand samples to other organizations and industries to examine our hypothetical models.

The second limitation concerns the study scales. Carmeli et al.'s (2010) scale that we used is a three-dimensional scale that contains openness, accessibility, and availability. However, because academia has not reached a unified consensus on the definition of inclusive leadership, different scholars have developed a variety of measurement questionnaires according to their own study results. Thus, these scales may not be able to reflect the overall picture of inclusive leadership. Moreover, all the questionnaires used are positive scoring questionnaires, so it is possible for the participants to guess the purpose of the measurement. Although we have adopted a longitudinal research design, which effectively avoids the impact of social desirability, it may not be able to eliminate its impact. In future studies, it is strongly suggested that a new measurement is compiled to generate a comprehensive understanding of inclusive leadership and that a combination of a questionnaire survey and an experiment is applied to enhance the credibility of the data.

## Data Availability

The raw data supporting the conclusions of this article will be made available by the authors, without undue reservation.
